# Arterial Calcification on Wrist Radiographs May Suggest Need for Evaluation of Atherosclerosis in Asymptomatic Individuals

**DOI:** 10.1155/2019/6156948

**Published:** 2019-07-03

**Authors:** Lauren E. Watchmaker, Jennifer M. Watchmaker, Greg P. Watchmaker

**Affiliations:** ^1^University of Wisconsin School of Medicine and Public Health, 750 Highland Ave, Madison, WI 53726, USA; ^2^Vanderbilt University School of Medicine, 1161 21st Ave S # D3300, Nashville, TN 37232, USA; ^3^The Milwaukee Hand Center, 1535 W. Market Street, Mequon, WI 53092, USA

## Abstract

Asymptomatic individuals with significant coronary artery disease (CAD) are at risk for unanticipated cardiac events including myocardial infarction (MI). Laboratory studies, stress tests, and coronary artery imaging including coronary artery calcium (CAC) scoring evaluate at-risk individuals. Hand and wrist x-rays demonstrating significant arterial wall calcification may provide an additional means to identify asymptomatic individuals at risk for cardiac events. Here we report a case series of patients without known cardiac disease who demonstrated significant calcium deposits in the radial and/or ulnar arteries in radiographs performed for evaluation of their hand conditions. Each series patient was subsequently found to have calcification on coronary artery imaging and an elevated risk of future cardiac events. Our series suggests that peripheral arterial calcifications observed by radiologists and hand specialists may warrant systemic evaluation for atherosclerosis in other areas of the body.

## 1. Introduction

Every year, about 635,000 Americans have their first heart attack [1]. Not all patients experiencing a cardiac event have prior known disease. The reason is multifactorial and includes individuals with risk factors who do not engage in healthcare and undergo screening tests as well as patients whose tests do not detect cardiac disease which is actually present. Traditionally, electrocardiograms, stress tests, and laboratory studies have been utilized as screening tools; however, more recently, noncontrast computed tomography imaging of the coronary arteries with image acquisition gated to the patient's electrocardiogram has made quantification of coronary artery calcification possible. Coronary artery calcium (CAC) scoring has been demonstrated to not only show current coronary disease but also predict future cardiac events [2, 3]. Budoff* et al*. studied a cohort of 25,253 asymptomatic patients and found that even mildly elevated CAC scores were associated with a 6-fold increase in CAD, and higher CAC scores were associated with up to a 62-fold increased risk of cardiac events within 10 years [4].

Radiologists and hand specialists engage with a broad demographic of patients from newborns with congenital disease to elderly patients with arthritis and fractures. This demographic includes young and middle-aged adults who may be unaware of evolving coronary pathology. Although older individuals with long-standing diabetes are known to develop arterial wall calcification visible on plain radiographs, younger individuals and nondiabetics can also develop such calcifications. The association between peripheral calcification and CAD has already been demonstrated for lower extremity vessels [5]. Following Institutional Review Board review and exemption, we collected a case series of patients with peripheral arterial calcifications on upper extremity radiographs who underwent subsequent CAC testing and report these findings.

## 2. Cases

**Figure 1:**
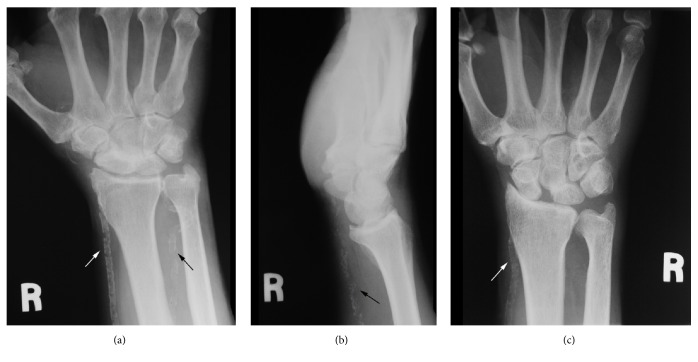
(a) Patient 1 with both radial artery (white arrow) and ulnar artery (black arrow) calcification. (b) Patient 2 with ulnar artery calcification. (c) Patient 3 with radial artery calcification.

Patient 1, a 59 year-old male with no past medical history, presented with wrist pain. An x-ray study of the right wrist demonstrated advanced arthritis in his area of symptoms, but also extensive calcification of both radial and ulnar arteries (Figure 1(a)). Based on this finding, further discussion regarding the presence of peripheral vascular disease and cardiac disease was undertaken. The patient denied any personal history of symptoms or previous cardiac workup. It was suggested that the patient considers further evaluation including a CAC score. The scan identified a total coronary calcium score of 424 which indicates extensive plaque burden. Based upon age-adjusted population data, this patient is at a high risk of a future cardiac event.

Patient 2, a 60 year-old otherwise healthy man, presented with pain in his right wrist which developed acutely when lifting a heavy machine. The x-ray study demonstrated scapholunate widening and radiocarpal arthrosis in addition to significant calcification of his ulnar artery which is most clearly seen on the lateral radiograph (Figure 1(b)). Similar to Patient 1, additional history revealed no prior symptoms or workup for CAD. A CAC score of 174 was interpreted as evidence of moderate calcium plaque with possible areas of significant narrowing. All of the calcifications were found in the left anterior descending artery.

Patient 3, a 59 year-old male with a 37-year history of diabetes but no known cardiac disease, also presented with wrist pain prompting an x-ray demonstrating scapholunate advanced collapse as well as arterial calcification (Figure 1(c)). A CAC score of 1412 indicated significant coronary disease.

## 3. Discussion

In our series of patients with upper extremity calcification, each patient without known cardiac disease was subsequently found to have an elevated CAC score indicating an increased risk for future cardiac events. Radiologists and hand specialists evaluate radiographs in an age demographic that is at risk for unrecognized CAD. Blais* et al*. found that younger individuals with CAD were more likely than older individuals to have sudden death from an MI as the initial presentation [6]. In the lower extremity, Lehto* et al. *in a large series of non-insulin-dependent diabetic patients found that femoral calcification carried a 1.6x risk of cardiac mortality and strong association of future cardiac events over a seven-year period [7]. In histologic examination of vascularly compromised extremities, O'Neil* et al.* demonstrated that significant medial calcification in addition to intimal thickening lead to narrowing of the vessel and in some instances complete obliteration of the lumen [8].

Radiographically visible arterial calcifications are more prevalent in patients with chronic kidney disease (CKD) and diabetes [9, 10]. This has clinical implications since the radial artery is commonly used as a conduit for coronary artery bypass (CAB) and presence of calcifications may reduce suitability of this graft [11, 12]. Deshpande* et al.* found that 4 patients in their series of 130 had clinically significant radial artery calcification that precluded its use for grafting [13].

Vessel wall calcifications that occur as sheet-like layers within the media of the wall are referred to as Monckeberg's Sclerosis [14]. Medial calcifications reduce arterial wall compliance and have been associated with increased mortality when involving large vessels [15]. Intimal calcifications, by contrast, narrow the arterial lumen more directly and are more likely associated with dyslipidemia and atherosclerotic plaques [14]. Although peripheral calcifications are not uncommon in CKD and diabetic patients, such radiographic findings in the younger or nondiabetic patient may be an important finding to discuss with the patient and report back to their primary care physician.

Additional research as to the strength of the association between peripheral calcification and future cardiac events will help define this relationship and need for additional workup. Based on this case series, we suggest that incidental findings of radial and/or ulnar artery calcification on routine radiographs may warrant systemic evaluation for atherosclerosis in other areas of the body.
